# Quantitative comparison of the mRNA content of human iPSC‐derived motor neurons and their extracellular vesicles

**DOI:** 10.1002/2211-5463.13059

**Published:** 2021-02-02

**Authors:** Kentaro Otake, Keiko Adachi‐Tominari, Hiroaki Nagai, Masayo Saito, Osamu Sano, Yoshihiko Hirozane, Hidehisa Iwata

**Affiliations:** ^1^ Innovative Biology Laboratories Neuroscience Drug Discovery Unit Research Takeda Pharmaceutical Company Limited Fujisawa Japan

**Keywords:** biomarker, exosome, extracellular vesicle, induced pluripotent stem cell, motor neuron, RNA sequencing

## Abstract

Extracellular vesicles (EVs) contain various cargo molecules, including RNAs and proteins. EVs, which include exosomes, are predicted to be suitable surrogates of their source cells for liquid biopsy to measure biomarkers. Several studies have performed qualitative comparisons of cargo molecule repertoires between source cells and their EVs. However, quantitative comparisons have not been reported so far. Furthermore, many studies analyzed microRNAs or proteins in EVs, but not mRNAs. In this study, we analyzed mRNAs in motor neurons and their EVs. Normal human‐induced pluripotent stem cells were differentiated into motor neurons, and comprehensive analysis of mRNAs in the cells and their EVs was performed by RNA sequencing. Differential analysis between cellular and EV mRNAs was performed by edgeR after normalization of read count. The results suggest that signatures in the abundance of EV mRNAs were different from those of cellular mRNAs. Comparison of intracellular vesicle and EV mRNA abundance showed negatively and positively biased genes in the EVs. Gene Ontology analysis revealed that the genes showing negatively biased abundance in the EVs were enriched in many functions regarding neuronal development. In contrast, the positively biased genes were enriched in functions regarding cellular metabolism and protein synthesis. These results suggest that mRNAs in motor neurons are loaded into EVs to regulate certain mechanisms, which are yet to be elucidated.

## Graphical abstract

Extracellular vesicles (EVs) are secreted from various cell types and carry cargo derived from their source cells. However, the quantitative relationship between the cargos of EVs and that of their source cells is unclear, especially for mRNA. To elucidate this, we compared comprehensive RNA expression profiles using induced pluripotent stem cell‐derived motor neurons and their secreted EVs.

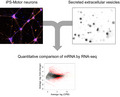

**Abbreviations**
ALS: amyotrophic lateral sclerosis
ChAT: choline acetyltransferase
CPM: counts per million mapped reads
CSF: cerebrospinal fluid
Ct: cycle threshold
D‐PBS: Dulbecco's phosphate‐buffered saline
eGFP: enhanced green fluorescence protein
ER: endoplasmic reticulum
EV: extracellular vesicle
FDR: false discovery rate
GO: Gene Ontology
iPSC: induced pluripotent stem cell
MNP: motor neuron progenitor
NEP: neuroepithelial progenitor
NTA: nanoparticle tracking analysis
RA: retinoic acid
RNA‐seq: RNA sequencing
RT‐qPCR, reverse transcription‐quantitative: PCR
SD: standard deviation

Exosomes are known as a representative subset of extracellular vesicles (EVs) that are secreted from any type of cell [1]. Exosomes are secreted from the cells via the formation of multivesicular bodies in endosomes and their fusion to the plasma membrane [2]. Microvesicles are also a subset of EVs, and they are secreted by a budding of plasma membrane [3]. The size of exosomes ranges from 30 to 200 nm, while that of microvesicles ranges from 100 to 1 μm [4]. However, it is difficult to completely separate and distinguish exosomes from other EV subsets because their diameters, marker molecules and cargos are rather overwrapped. Therefore, EVs are used as a general term [5]. EVs carry functional cargo molecules, such as RNAs and proteins, inside and outside of their membranes [6]. Generally, cargo molecules in EVs are considered to reflect at least a portion of components of the source cells. Accordingly, EVs can theoretically be surrogate materials of cells, tissues and organs for measurement of biomarkers in a liquid biopsy of biofluids.

We previously reported development of a methodology for EV mRNA sequencing [7]. Using this technique, we identified several mRNAs that were increased or decreased in cerebrospinal fluid (CSF) EVs from amyotrophic lateral sclerosis (ALS) in comparison with those from normal healthy donors. ALS is a serious neurodegenerative disease characterized by the loss of motor neurons [8]. Ideally, these changes in CSF‐EV mRNAs are expected to be derivable from motor neurons for use as surrogate biomarkers. In reality, it is difficult to analyze which tissues or cell types contributed to changes in CSF EVs, because miscellaneous EVs are secreted from various types of cells throughout the body in biofluids. Simplified *in vitro* analysis with pure culture system would help us to understand to what degree the cargo molecules in EVs resemble a state of motor neurons. To investigate direct relationships between cargo molecules in EVs and those of their source cells, induced pluripotent stem cells (iPSCs) are considered to be a powerful tool [9], especially for human background. Many efforts have been made regarding differentiation of human iPSCs into specific cell types, such as motor neurons and dopaminergic neurons [10]. These technologies enable study of physiological roles of specific cell types and cellular or molecular dysfunction with some stimuli to recapitulate disease conditions in a dish.

Although the relationship of cargo molecule repertoires between the source cells and their EVs was already reported in a few cases [11, 12], there is no report regarding quantitative comparison of cargo molecules, particularly mRNA. In this study, we aimed to answer a simple question: whether the same amount of EV mRNAs exists as in the source cells. As models of motor neurons, several iPSCs established from normal human, ALS patient and genome‐edited cells were efficiently differentiated into motor neurons. RNA sequencing (RNA‐seq) was performed against RNAs extracted from motor neurons and their EVs in the culture supernatant. The abundance of each mRNA and the presence of functional enrichment between them were clarified.

## Materials and methods

### Preparation of iPSC‐derived motor neurons

201B7, a healthy control iPSC line obtained from Kyoto University [8], and iPSCs from an ALS patient harboring mutated *TDP‐43* at G298S (ND50007; RUCDR Infinite Biologics, Piscataway, NJ, USA) were maintained in Stemfit AK02N (Ajinomoto Co., Inc., Tokyo, Japan) medium on a plate coated with iMatrix‐511 (FUJIFILM Wako Pure Chemical Corporation, Osaka, Japan). For differentiation, these cells were grown to about 80% confluency and dissociated by EDTA (Thermo Fisher Scientific, Waltham, MA, USA). The cells were then differentiated into motor neuron progenitors (MNPs) and finally maturated to motor neurons by previously reported methods [13, 14, 15]. In brief, multipotent neuroepithelial progenitors (NEPs) were induced from iPSCs for 6 days in the presence of CHIR99021 (FUJIFILM Wako Pure Chemical Corporation) at 3 μm, and DMH‐1 (MilliporeSigma, Burlington, MA, USA) and SB431542 (FUJIFILM Wako Pure Chemical Corporation) both at 2 μm. Initial MNPs were induced from NEPs for 6 days in the presence of CHIR99021 at 1 μm, DMH‐1 at 2 μm, SB431542 at 2 μm, retinoic acid (RA) (FUJIFILM Wako Pure Chemical Corporation) at 0.1 μm and Purmorphamine (MilliporeSigma) at 0.5 μm. MNP‐neurospheres were further induced for an additional 5 days in the presence of RA at 0.5 μm and Purmorphamine at 0.1 μm. MNP‐neurospheres were dissociated, frozen and stored in liquid nitrogen until starting experiments for maturation into motor neurons. Upon differentiation to motor neurons, frozen MNPs were recovered and maintained in the medium containing RA at 0.5 μm, Purmorphamine at 0.1 μm and Compound E (MilliporeSigma) at 0.1 μm. The essential medium was used throughout all steps, and it was composed of Dulbecco's modified Eagle's medium/F12 (FUJIFILM Wako Pure Chemical Corporation), Neurobasal Medium (Thermo Fisher Scientific Inc.), N2 Supplement (FUJIFILM Wako Pure Chemical Corporation), B27Supplement (Thermo Fisher Scientific Inc.), ascorbic acid (MilliporeSigma), GlutaMAX (Thermo Fisher Scientific Inc.) and penicillin–streptomycin solution (FUJIFILM Wako Pure Chemical Corporation).

In addition, we used iPSC‐derived motor neurons with normal *TDP‐43* and genome‐edited *TDP‐43* at M337V (FUJIFILM Cellular Dynamics, Inc., Madison, WI, USA) according to the manufacturer's instructions. All experiments were performed under approval of the ethical review board of Shonan Research Center of Takeda Pharmaceutical Company Limited.

### Immunocytochemistry

201B7 MNPs were recovered from frozen stock and seeded to a 384‐well plate (CellCarrier Ultra; PerkinElmer, Inc., Waltham, MA, USA) coated with Matrigel (Corning Incorporated, Corning, NY, USA) at 1.5 × 10^4^ cells per well. Medium change was performed every 2 or 3 days. At day 7, the cells were fixed by addition of 4% paraformaldehyde (FUJIFILM Wako Pure Chemical Corporation) for 30 min and permeabilized by the addition of Dulbecco's phosphate‐buffered saline (D‐PBS) (–) (FUJIFILM Wako Pure Chemical Corporation) containing 0.4% Triton X‐100 and 1% normal goat serum (both from FUJIFILM Wako Pure Chemical Corporation) for an hour. Only in the double staining of Tuj1 and choline acetyltransferase (ChAT) was Pierce Protein‐free T20 Blocking Buffer (Thermo Fisher Scientific Inc.) used in blocking and antibody dilution processes instead of 1% normal goat serum. The cells were then incubated with anti‐Tuj1 (Clone D719; at a dilution of 1 : 200; Cell Signaling Technology, Danvers, MA, USA), anti‐SMI‐32 (Clone SMI 32; 1 : 1000; BioLegend, Inc., San Diego, CA, USA), anti‐Islet1 (39.4D5; 1 : 50; Developmental Studies Hybridoma Bank, Iowa City, IA, USA) and anti‐ChAT (Clone AB144P; 1 : 100; MilliporeSigma) for 5 h at room temperature. After washing with D‐PBS (–), the cells were incubated with Alexa Fluor 488‐conjugated anti‐rabbit IgG and Alexa Fluor 568‐conjugated anti‐mouse IgG (1 : 1000; all from Thermo Fisher Scientific Inc.), together with DRAQ7 (1 : 150; Abcam, Cambridge, UK) or NucSpot Live 650 (1 : 20 000; Biotium, Fremont, CA, USA), for 1 h at room temperature. In the experiment to detect HB9 promotor activity by means of enhanced green fluorescence protein (eGFP), the cells were infected with LV‐HB9‐GFP (VB200604‐1088etr; VectorBuilder Inc., Chicago, IL, USA) [16, 17] at a multiplicity of infection of 10 on day 2. The cells were fixed as described earlier on day 7. Stained cells were stored at 4 °C until obtaining images by CV7000 Cell Voyager imaging apparatus (Yokogawa Electric Corporation, Kanazawa, Japan) with 60× magnification. Image analysis was performed with cv7000 Analysis Software (Yokogawa, Japan) using 60× magnified images. Nuclei were defined by its size larger than 20 μm^2^, and condensed nuclei were excluded. Next, the fluorescence intensity of each channel within the nucleus was quantified to set a threshold to distinguish positive cells from negative cells. In regards to immunostaining of Islet1 and ChAT, the intensity threshold was set to 150 (a.u.) and that of Tuj1 was 270 (a.u.) to assure clear separation between negative and positive staining, in contrast with nuclear‐only staining. Also, that of SMI‐32 was 140 (a.u.). In the detection of HB9‐eGFP fluorescence, the intensity threshold was set to 40 (a.u.), and that of Tuj1 was set to 150 (a.u.) (Fig. S1). These conditions were applied uniformly to all six replicates.

### Preparation of cellular RNAs and EV RNAs

MNPs were recovered from frozen stock and seeded to a generic six‐well plate coated with Matrigel at 1 × 10^6^ cells per well. Medium change was performed every 2 or 3 days. At day 7, the entire volume of medium was gently replaced with 3 mL of fresh medium, and the cells were further cultured for 72 h to ensure a sufficient amount of EVs could be accumulated in the culture supernatant. At day 10, the entire volume of the culture supernatant was collected and passed through a filter with a pore size of 0.8 μm (MilliporeSigma). The remaining motor neurons at the bottom of wells were homogenized with 700 μL QIAzol, supplied with miRNeasy Mini Kit (QIAGEN, Hilden, Germany), after collection of culture supernatant. Chloroform (FUJIFILM Wako Pure Chemical Corporation) with a volume of 140 μL was added to the homogenate. Total RNA was then extracted in accordance with the instructions of the miRNeasy Mini Kit. The yield and quality of total RNA were measured using Qubit RNA HS Assay Kit (Thermo Fisher Scientific Inc.) and Agilent RNA6000 Nano Kit (Agilent Technologies Inc., Santa Clara, CA, USA), respectively. EV RNAs were purified from the filtered culture supernatant with exoRNeasy Maxi Kit (QIAGEN) in accordance with the manufacturer's instruction.

### Nanoparticle tracking analysis and western blotting for EVs

To isolate intact EVs, we applied exoEasy Maxi Kit (QIAGEN) for filtered culture supernatant of 201B7‐motor neurons at day 10 instead of miRNeasy Maxi Kit used for RNA preparation. This was because intact EVs are disrupted by a direct addition of lysis buffer to EV‐trapped filter when using miRNeasy, and they adopt the same spin filter to trap EVs [18]. The eluate from exoEasy was buffer exchanged to D‐PBS (–) by Vivacon‐500 100‐kDa molecular weight cutoff spin column (Sartorius, Gottingen, Germany). Subsequently, it was subjected to nanoparticle tracking analysis (NTA) using NanoSight NS500 (Malvern Instruments, Malvern, UK). Particle diameter was calculated from the velocity of Brownian motion.

The isolated EVs and their source cells 201B7‐motor neurons were lysed in M‐PER Mammalian Protein Extraction Reagent (Thermo Fisher Scientific Inc.). Protein concentration was determined using micro BCA Protein Assay Kit (Thermo Fisher Scientific Inc.). The lysates containing 50 ng protein were mixed with 4× NuPAGE LDS sample buffer and 10× Sample Reducing Agent (Thermo Fisher Scientific). The mixture was incubated at 70 °C for 10 min and subjected to gel electrophoresis with the NuPAGE SDS/PAGE system (Thermo Fisher Scientific) with protein size standard (Bio‐Rad Laboratories, Hercules, CA, USA) and blotted to polyvinylidene difluoride membrane (Bio‐Rad Laboratories). The blot was blocked with polyvinylidene difluoride Blocking Reagent for Can Get Signal (TOYOBO, Osaka, Japan). Anti‐Flotillin‐1 (BD Biosciences, Franklin Lakes, NJ, USA) and anti‐Calnexin (Abcam, Cambridge, UK) were used in 1000‐fold dilution in Can Get Signal solution 1. Horseradish peroxidase‐conjugated anti‐mouse secondary antibodies (Cell Signaling Technology) were used with dilution of 5000‐fold in Can Get Signal solution 2. The luminescence was developed by SuperSignal West Femto Maximum Sensitivity Substrate (Thermo Fisher Scientific), and the images were obtained by Amersham Imager 680 (GE Healthcare, Chicago, IL, USA).

### Library preparation for EV mRNA‐seq

To prepare a library for RNA‐seq, we used SMART‐seq v4 Ultra Low Input RNA Kit for Sequencing (Takara Bio Inc., Shiga, Japan). For cellular mRNAs, the input amount of RNA was 1 ng. For EV mRNAs, the input volume of RNA was adjusted to 9.5 μL, which is the maximal volume described in the instructions, because concentrations of EV mRNAs were under the detection limit. Amplification of cDNA was performed over 10 and 16 cycles for cellular mRNAs and EV mRNAs, respectively. The concentration of purified cDNA was measured with the Qubit dsDNA HS Kit (Thermo Fisher Scientific Inc.). Illumina sequencing adaptors and indices were ligated to fragmented DNA by NextEra XT DNA Library Prep Kit (Illumina Inc., San Diego, CA, USA) with input DNA in the amount of 150 and 1 ng for cellular mRNAs and EV mRNAs, respectively. Size distribution and concentration of the prepared library were evaluated by Agilent High Sensitivity DNA Kit (Agilent Technologies Inc.) and Qubit dsDNA HS Kit, respectively.

### Next‐generation sequencing

Paired‐end sequencing for 76 bp was run on the NextSeq 500 system with NextSeq 500/550 High Output Kit (Illumina Inc.). Each read in the generated FASTQ file was mapped to human genome reference B37.3 with the aid of RNA‐seq pipeline implemented in OmicSoft ArrayStudio (QIAGEN). Relative expression of EV mRNAs was calculated as counts per million mapped reads (CPM). Genes with averaged CPM less than 10 were removed. Paired panels of correlation in CPM among samples were depicted by psych package implemented in r (distributed by CRAN) under operation of its integrated development environment RStudio.

### Statistical analysis

For the differential analysis of mRNAs between motor neurons and their EVs, edgeR package implemented in r was used. In this pipeline, adjusted *P*‐value for a false discovery rate (FDR) correction was performed by Benjamini–Hochberg method. With the differentially abundant mRNAs with adjusted *P* < 0.05, Gene Ontology (GO) analysis was performed using metacore (Clarivate Analytics, Philadelphia, PA, USA) version 19.3.69800. The entire list of the differentially abundant mRNAs and GO processes with statistical values is shown in Tables S1 and S2, respectively.

### Quantitative PCR

First, to evaluate a population of differentiated cells, we performed probe‐based reverse transcription‐quantitative PCR (RT‐qPCR) using RNA extracted from undifferentiated iPSCs and differentiated motor neurons. RNA input of 100 ng was reverse transcribed by SuperScript VILO IV Master Mix (Thermo Fisher Scientific, Inc.) according to the manufacturer. The expression levels of *ISL1*, *MNX1*, *NEFH*, *GFAP*, *SLC1A3*, *AIF1*, *ITGAM*, *MOG* and *ACTB* were measured. *ACTB* was used for normalization of cycle threshold (Ct) values between both cells as an internal control.

Second, to confirm differentially abundant genes, we performed probe‐based RT‐qPCR. For the cellular RNA, RNA input of 100 ng was reverse transcribed as described earlier, whereas the maximum volume of RNA (8 μL RNA in total 10 μL scale) was subjected for EV RNA. *ISL1* and *NEFH* were used as less abundant genes, whereas *RPS2* and *RAB13* were used for more abundant genes in EVs compared with the cells based on the result of RNA‐seq (Table 1). *ACTB* was used for normalization of Ct values between the cells and their EVs.

**Table 1:** Differentially abundant genes between iPSC‐derived motor neurons and their EVs. Fold change (FC) and CPM are represented as base‐2 logarithms with raw *P*‐values and adjusted *P*‐values representing FDR.

Gene	logFC	logCPM	*P*‐value	FDR
*GNG5*	8.38	8.15	4.24E−94	6.62E−90
*VIM*	6.62	11.01	1.79E−87	1.40E−83
*UBE2C*	9.35	7.77	5.55E−87	2.89E−83
*CDC20*	10.36	6.90	2.42E−82	9.47E−79
*PCDHGB6*	−11.86	6.95	8.24E−73	2.58E−69
*HNRNPH2*	−14.92	6.68	3.66E−69	9.55E−66
*BIRC5*	8.57	6.59	1.08E−68	2.41E−65
*AURKB*	11.27	6.60	2.44E−67	4.78E−64
*TTYH1*	6.54	8.15	1.17E−66	2.04E−63
*HIST1H4C*	5.40	10.30	6.97E−66	1.09E−62
*APP*	−5.79	7.99	4.47E−64	6.36E−61
*KAL1*	−6.26	7.89	1.64E−62	2.14E−59
*MKI67*	9.76	6.22	2.37E−60	2.85E−57
:	:	:	:	:
*RPS2*	4.61	12.64	4.52E−55	3.93E−52
*RPS11*	4.58	11.07	8.56E−55	7.05E−52
*RAB13*	7.23	6.68	1.28E−54	1.00E−51
:	:	:	:	:
*TUBB3*	1.25	11.04	1.07E−6	9.77E−5
:	:	:	:	:
*ISL1*	−5.82	4.71	1.75E−6	1.50E−5
:	:	:	:	:
*KIF5C*	−1.66	8.66	7.88E−6	5.72E−5
:	:	:	:	:
*NEFH*	−2.07	3.56	0.02	0.04

In both examinations, mixtures of primers and probes were synthesized and purchased from Integrated DNA Technologies (Coralville, IA, USA). The assay IDs were listed in Table 2. Preamplification of cDNA was performed with TaqMan PreAmp Master Mix (Thermo Fisher Scientific, Inc.) in accordance with the instructions of the manufacturer. Primers and probes, TaqPath qPCR Master Mix (Thermo Fisher Scientific, Inc.) and preamplified cDNA were mixed, and qPCR was performed on Applied Biosystems Quant Studio 7 Flex Real‐Time PCR System (Thermo Fisher Scientific, Inc.). The Ct value was calculated from the amplification curve with automatic threshold and baseline condition. For a correction of multiple comparison in gene expressions, FDR was set to 0.05.

**Table 2:** RT‐qPCR probes to verify induction of motor neuron. All probes were predesigned and available from Integrated DNA Technologies. Assay IDs are shown.

Gene	Marker	Assay ID
*ISL1*	Neuron	Hs.PT.58.2143768
*MNX1*	Neuron	Hs.PT.58.20467334
*NEFH*	Neuron	Hs.PT.58.4224792
*GFAP*	Astrocyte	Hs.PT.58.14980282
*SLC1A3*	Astrocyte	Hs.PT.58.2436320
*AIF1*	Microglia	Hs.PT.56a.40712748.gs
*ITGAM*	Microglia	Hs.PT.58.40141028
*MOG*	Oligodendrocyte	Hs.PT.58.14576268
*RPS2*	–	Hs.PT.58.22843181
*RAB13*	–	Hs.PT.58.23195734

## Results

### Preparation of iPSC‐derived motor neurons

**Fig. 1:**
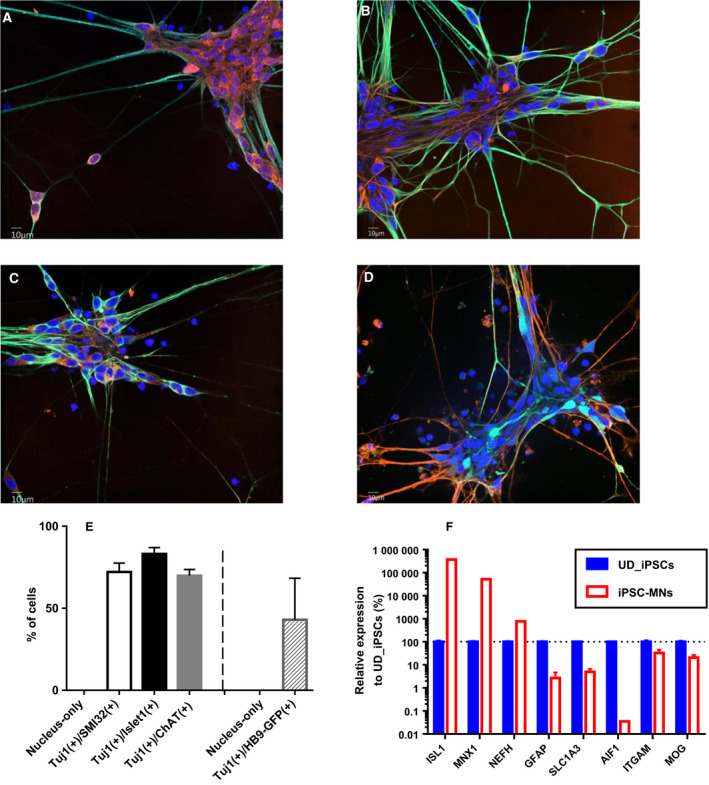
Preparation of human iPSC‐derived motor neurons. Human iPSCs were differentiated to motor neurons. (A–C) Green and blue colors represent the immunostaining of Tuj1 and DRAQ7‐stained cellular nuclei, respectively. Orange color represents Islet1 (A), SMI‐32 (B) and ChAT (C), respectively. The image was obtained at day 7 after seeding of MNPs at original magnification of 60×. Scale bars: 10 μm. (D) Green, orange and blue colors represent the fluorescence of eGFP, immunostaining of Tuj1 and NucSpot Live 650‐stained cellular nuclei, respectively. The image was obtained at day 7 after seeding of MNPs at original magnification of 60×. Scale bar: 10 μm. (E) Purity of each population was analyzed according to the method described in the Materials and Methods. The average of six replicated wells is represented. The error bar indicates SD of *n* = 6. (F) The expression levels of cell‐type‐specific genes were investigated by RT‐qPCR and normalized by ACTB. Those in 201B7 iPSCs‐derived motor neurons (MNs) were compared with those in undifferentiated (UD) iPSCs. The average of three replicates is represented, and the bar indicates SD of *n* = 3.

As a representative of normal human iPSCs, 201B7 cells were differentiated into NEPs, MNPs and motor neurons in a stepwise manner, as described in a section of Materials and methods. Motor neurons cultured for 7 days after seeding of MNPs showed a neural network with frequent expression of Tuj1, Islet1 (Fig. 1A), SMI‐32 (Fig. 1B) and ChAT (Fig. 1C). Furthermore, HB9 promotor activity was confirmed by means of the expression of eGFP (Fig. 1D). The population positive for both Tuj1 and Islet1 was 83.1% on average with standard deviation (SD) of 3.9% in six replicated wells at day 7 (Fig. 1E). That for both Tuj1 and SMI‐32 was 72.1% with 5.4% SD. The positive rate for both Tuj1 and ChAT showed 69.8% with 3.8% SD. In addition, the double‐positive population for Tuj1 and HB9‐GFP was 43.1% with 25.3% SD. To investigate the purity of the motor neuronal population against other cell‐type populations, we performed RT‐qPCR in comparison with undifferentiated iPSCs. *ISL1*, *MNX1* and *NEFH* that encode Islet1, HB9 and SMI‐32, respectively, were used as neuronal marker genes. *GFAP* and *SLC1A3* were used as astrocytic genes. *AIF1* and *ITGAM* were used as microglial genes. *MOG* was used as an oligodendrocytic gene. Motor neurons at day 10 showed much higher gene expression of *ISL1*, *MNX1* and *NEFH* than those in undifferentiated iPSCs (Fig. 1F). Contrary to neural genes, all astrocytic, microglial and oligodendrocytic genes were less expressed in motor neurons than those in undifferentiated iPSCs.

### Characterization of isolated EVs

**Fig. 2:**
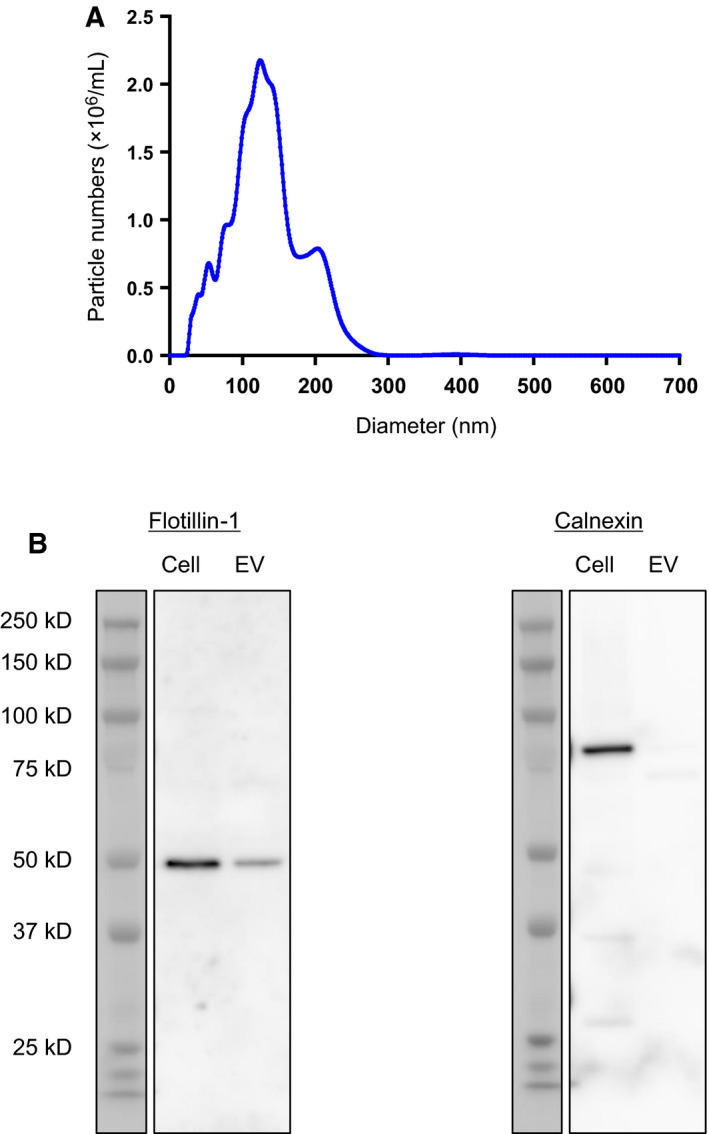
Characterization of EVs. The eluate from exoEasy started from cell culture supernatant of 201B7 iPSC‐derived motor neurons was analyzed for (A) NTA and (B) western blotting against Flotillin‐1 and calnexin. Total protein amounts of cell lysate and EV lysate were equally adjusted to 50 ng to be loaded on the gels.

The eluate from exoEasy started from cell culture supernatant of 201B7 iPSCs‐derived motor neurons was buffer exchanged to D‐PBS (–) and analyzed for their diameter by NTA. The nanoparticles showed 133 nm in their averaged diameter, and the most frequent size was 124 nm (Fig. 2A). As a probe of EVs, the presence of Flotillin‐1 was observed both in cell lysate and EVs in western blotting (Fig. 2B). To eliminate the possibility of contamination with other membrane organelles, such as endoplasmic reticulum (ER), the absence of calnexin in EVs was confirmed (Fig. 2B). Taken together with NTA and western blotting, the eluate from exoEasy applied with cell culture supernatant of 201B7 iPSC‐derived motor neurons contained EVs without contamination of the ER membrane.

### Reproducibility of transcriptomic analysis

**Fig. 3:**
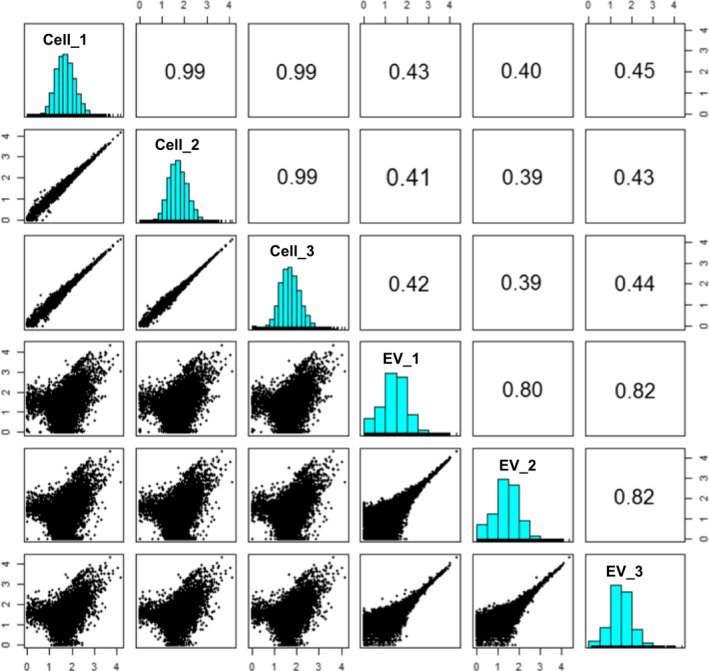
Paired panels of correlation in gene expression among samples. CPM of each gene in a sample is plotted and compared with the others in the window. The Pearson's product‐moment correlation coefficient is described in the oppositely plotted window. Digits along the axes of windows represent logarithmic values of CPM.

Short reads generated from a next‐generation sequencer were mapped to the human genome reference, and raw count data for each gene were calculated. Raw counts for each gene were then converted to CPM to adjust library size among samples. Both cellular mRNAs and EV mRNAs were examined in three biological replicates (*n* = 3). Low‐abundance genes with averaged CPM less than 10 were excluded from analysis to obtain a robust result. As a result, 9849 genes were used for downstream analysis. Abundance of each gene represented by CPM was compared with the others. Samples for cellular mRNAs showed high reproducibility among three biological replicates (upper‐left windows in Fig. 3). The Pearson's product‐moment correlation coefficient was 0.99 in all samples. Samples for EV mRNAs also showed high reproducibility, although lower than those in cellular mRNAs (lower‐right windows in Fig. 3). The Pearson's product‐moment correlation coefficient was approximately 0.8 in all samples. Genes with CPM less than 100 (2 on logarithmic scale) tended to show some variability in their abundance among biological replicates. This was possibly due to a low concentration of EV mRNAs, which was under the lower limit of quantification (data not shown). Although the RNA‐seq technology adopted here was highly sensitive, there was a technical limitation, especially for genes of relatively low abundance. The high reproducibility among biological replicates in each group assured technical reliability of RNA‐seq. In comparison, between cellular mRNAs and EV mRNAs, a certain level of correlation was observed, with a correlation coefficient of approximately 0.4 (upper right and lower left in Fig. 3). The variance between pairs of groups was observed not only in the genes with CPM less than 100 but also in the genes with high CPM greater than 100. These distribution patterns suggested that the order in the abundance of EV mRNAs was not the same with that of cellular mRNAs.

### Identification of differentially abundant mRNAs and their functions

**Fig. 4:**
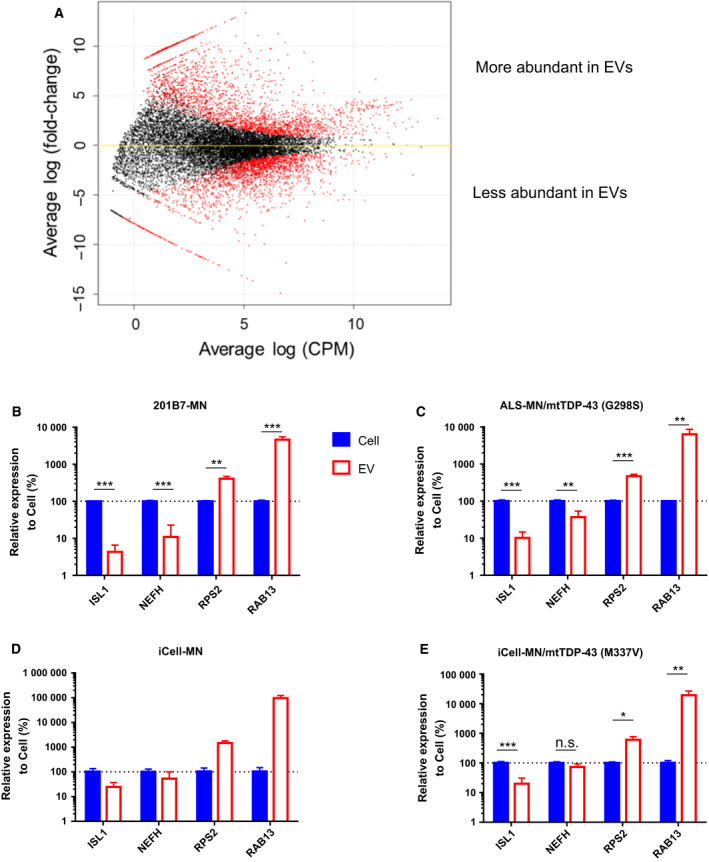
Differential analysis of gene expression between iPSC‐derived motor neurons and their EVs. (A) The result of differential analysis of gene expression by edgeR is visualized. The differentially expressed genes with adjusted *P* < 0.05 are colored in red. The representative differential abundance of *ISL1*, *NEFH*, *RPS2* and *RAB13* was confirmed by RT‐qPCR using several lines, (B) 201B7 iPSCs‐derived motor neurons, (C) ALS patient‐derived motor neurons harboring mutated TDP‐43 at G298S, (D) iCell motor neurons and (E) iCell motor neurons with genome‐edited TDP‐43 at M337V. The expression levels of these genes were normalized by *ACTB*. The data represent the average of *n* = 3 in (B), (C), and (E) and *n* = 2 in (D) with the error bar indicating SD. In statistical tests in (B), (C), and (E), *t*‐test without any assumption of variance was applied, and FDR for adjustment of multiple comparison was set to 0.05. The results were described as follows: ***P < 0.001, **P < 0.01, *P < 0.05; n.s., not significant. The statistical test was not performed in (D) because of insufficient number of replicates.

To further specify which genes were differentially abundant between the source cells and their EVs, we performed transcriptomic comparison between them using edgeR. First of all, genes with averaged raw count less than 10 were excluded from analysis to assure the result to be quantitative. The number of remaining genes to be analyzed was 15 638. In the differential analysis, trimmed mean of *M*‐value normalization was used to enable comparison among samples. As a result, 7657 genes showed raw *P* < 0.05. In FDR correction, 6603 genes showed adjusted *P* < 0.05 (Fig. 4A and Table S1). The number of genes that were less abundant was almost the same as those that were more abundant in EVs compared with the cells. In the EVs, 3378 genes were less abundant than those in their source motor neurons. The markers of motor neurons, such as *NEFH*, *ISL1* and *KIF5C* [19], were included in this group (Table 1). In contrast, 3225 genes were more abundant in the EVs. Particularly, mRNAs encoding ribosomal proteins RPLs and RPSs were enriched in the EVs (Table 1). In addition, mRNAs of histones, RhoGTPases, G‐protein coupled receptors and kinases were also concentrated in the EVs. Exceptionally, *TUBB3*, a neural precursor marker encoding Tuj1, was among the positively biased genes in the EVs. Probe‐based RT‐qPCR was performed to validate the differential abundance of *ISL1*, *NEFH*, *RPS2* and *RAB13* between the cells and EVs. The former two were representatives of less abundant genes, and the latter two were those of more abundant genes in EVs. Furthermore, to investigate whether this tendency was generic among different lines or specific to this line, several lines, including ALS patient‐derived iPSCs, were examined for these gene expressions. Ideally, RNA input amount for reverse transcription should be equally adjusted for all comparisons. However, it was not feasible because the concentration of EV RNA was under the quantification limit and lacked rRNA (data not shown). Therefore, based on the result of RNA‐seq, *ACTB* was chosen for normalization of Ct values because it was not significantly changed between the cells and EVs. Statistically significant lower abundance of both *ISL1* and *NEFH* and higher abundance of both *RPS2* and *RAB13* were observed commonly in 201B7 iPSC‐derived motor neurons and ALS patient‐derived motor neurons harboring mutated *TDP‐43* at G298S (Fig. 4B,C). In iCell motor neurons, we could prepare only two replicates, so that statistical test was not applied to this line. Although the tendency of biased abundance between the cells and EVs was almost the same with 201B7 iPSC‐derived motor neurons, the difference in *NEFH* abundance was not so large as seen in them (Fig. 4D). Similarly, the abundance in *NEFH* was not significantly different between the cells and EVs of iCell motor neurons with genome‐edited TDP‐43 at M337V, even though lower abundance of *ISL1* and higher abundance of both *RPS2* and *RAB13* were commonly observed (Fig. 4E). Thus, the consistency of RNA‐seq data was generally confirmed by RT‐qPCR in several lines except for small differences of *NEFH* abundance in iCell motor neuronal lines.

Next, the presence of functional enrichment in these differentially abundant genes between cellular mRNAs and EV mRNAs was investigated by GO analysis. Many functions associated with neuron development were extracted from genes showing negatively biased abundance in EVs (Tables 3 and S2). Also, the functions of localization and transport were observed in this group. In contrast, the functions regarding cellular metabolism and protein synthesis were extracted from genes showing positively biased abundance in the EVs (Tables 3 and S2).

**Table 3:** GO analysis of the differentially abundant genes. GO analysis was performed using MetaCore by Clarivate Analytics. Top 10 GO processes extracted from less abundant and more abundant genes in the EVs compared with their source motor neurons are represented with raw *P*‐values and adjusted *P*‐values representing FDR.

	Processes	*P*‐value	FDR
Less 1	Nervous system development	1.18E−71	1.22E−67
Less 2	Modulation of chemical synaptic transmission	6.33E−57	3.25E−53
Less 3	Regulation of transsynaptic signaling	1.04E−56	3.57E−53
Less 4	Localization	2.62E−56	6.74E−53
Less 5	Generation of neurons	2.28E−54	4.68E−51
Less 6	Neurogenesis	3.86E−54	6.61E−51
Less 7	Cellular localization	7.85E−53	1.15E−49
Less 8	Transport	9.70E−50	1.25E−46
Less 9	Establishment of localization	1.19E−49	1.36E−46
Less 10	Establishment of localization in cell	7.12E−49	7.31E−46
More 1	Cellular nitrogen compound metabolic process	3.56E−77	3.94E−73
More 2	Cellular component organization or biogenesis	2.66E−72	1.47E−68
More 3	Cellular process	1.91E−71	7.04E−68
More 4	Cellular nitrogen compound biosynthetic process	1.32E−68	3.37E−65
More 5	Cellular component organization	1.52E−68	3.37E−65
More 6	Cellular metabolic process	2.78E−68	5.13E−65
More 7	Biosynthetic process	4.58E−66	7.24E−63
More 8	Signal recognition particle‐dependent cotranslational protein targeting to membrane	4.26E−65	5.90E−62
More 9	Cellular biosynthetic process	1.41E−64	1.73E−61
More 10	Cotranslational protein targeting to membrane	3.36E−64	3.72E−61

## Discussion

We performed a comparison of mRNAs between EVs and their source cells using iPSC‐derived motor neurons. This was not only a comparison of repertoire but also included quantitative evaluation. We took advantage of an ability of RNA‐seq to quantify and compare mRNAs among different genes in both intragroups and intergroups after appropriate normalization with larger dynamic range than microarray. The validity of iPSC‐derived motor neurons was assessed by both immunocytochemistry and RT‐qPCR approaches (Fig. 1). As for the maturity of motor neurons, there were 83.1%, 69.8% and 43.1% positive populations for spinal motor neuronal markers, Islet1, ChAT and HB9, respectively (Fig. 1E). Especially, ChAT and HB9 are considered to be terminal spinal motor neuronal markers [20]. Therefore, we could assure the motor neurons that we used in this study showed a good differentiation efficiency and were heading for terminal maturation. As for the purity of motor neurons against other cell types, we could assure no or low contamination of them. It was because there was no increase in astrocytic, microglial and oligodendrocytic genes in the motor neurons compared with undifferentiated iPSCs (Fig. 1F). The validity of exoEasy column for the isolation of EVs secreted from iPSC‐derived motor neurons without contamination of the ER membrane was also shown in this study (Fig. 2B).

In the quantitative comparison of mRNAs between motor neurons and their EVs, a biased abundance of mRNAs in the EVs was observed (Figs 3 and 4). Particularly, it was notable that these biases were observed almost commonly in four lines, including ALS patient‐derived motor neurons (Fig. 4B–E), although they did not show axonal impairment in the normal culture conditions adopted in this study (data not shown). Given that the EVs completely reflected the contents of the source cells, the order in the abundance of mRNAs should be same between them. These biases strongly suggested that mRNAs in human iPSC‐derived motor neurons were not sorted to their EVs by chance without any biological mechanism. Although this is the first report to show the biased abundance of mRNAs in the EVs, these phenomena were also observed in the signature of microRNAs in cultured astrocytes [12]. It was reported that some RNA motifs play a role in the sorting of RNAs into EVs mediated by RNA‐binding proteins [21]. Difference in the localization of mRNAs and biogenesis of EVs may also affect the loading of mRNAs in their EVs. Generally, spliced mRNAs are exported from the nucleus and translated into proteins at cytosol. In neural cells, characteristic structures of soma and dendrites are formed. The transport of mRNAs from soma to dendrites or axon and consequent local protein synthesis at dendrites are often observed in neural cells [22]. As seen in Fig. 1, the motor neurons showed neurite outgrowth and neural networks. In contrast, biogenesis of EVs is considered to be dependent on the formation of endosomal multivesicular bodies [2] at soma.

It was demonstrated that a lot of neural markers, including markers of motor neurons (e.g., *NEFH* and *ISL1*), were included in a group of negatively biased genes in EVs. Although we observed the protein expression of motor neuron marker SMI‐32 at day 7 and elevated gene expression of *ISL1*, *MNX1* and *NEFH* at day 10 after seeding of MNPs, there remained a possibility of further maturation. This inability to assure a complete maturation of motor neurons was a limitation in this study. This was partially because of technical difficulties in maintaining motor neurons for a long period in two‐dimensional culture. In the course of neural maturation, neural genes might play important roles, and loading to EVs might be difficult in some mechanisms to be solved. Although we focused on only mRNAs in this study, whether these genes are also present in the EVs in a form of proteins is to be investigated. Regarding positively biased genes, enrichment of fundamental functions in the maintenance of cellular activity was observed. Because EVs are considered to be a communication tool for the cells, these mRNAs might exert some functions in other recipient cells. To date, there are many studies reporting functional improvement of recipient cells in some tissues on addition of EVs, especially those derived from mesenchymal stem cells [23]. The biological activity of neuron‐derived EVs as a communication tool in a healthy condition has not been reported so far. In neurodegenerative diseases, neuron‐derived EVs are suggested to eliminate misfolded and aggregated proteins, such as tau [24], α‐synuclein [25] and TDP‐43 [26], from the cells. Ironically, these mechanisms to reduce the burden of toxic proteins are also considered to contribute to the propagation of the toxic proteins, such as those with prion‐like characteristics.

In conclusion, we demonstrated that signatures in the abundance of EV mRNAs were not the same as those of cellular mRNAs, and they were biased according to the functions. In contrast, from a qualitative aspect, almost all mRNAs detected in the source cells could be also detected in their EVs. These results are based on a comparison of cellular and EV mRNAs in a natural state without any phenotypic abnormality in motor neurons, and they may serve as basic knowledge. Further investigation regarding the alteration of both cellular and EV mRNAs by some interventions (e.g., drug treatment) will consolidate the value of analyzing EVs as surrogate materials of motor neurons. In addition, upon adopting an optimal condition that disease iPSCs or induction of ALS‐causative mutations expresses phenotypic abnormality in differentiated motor neurons, discovery of disease‐specific biomarkers is of great interest in parallel with analysis of human biofluids such as CSF.

## Conflict of interest

All authors are employees of Takeda Pharmaceutical Company Limited.

## Author contributions

KO and YH contributed to the design of EV‐related research study. KO contributed to generation and analysis of data and writing of the manuscript. KA‐T, HN, MS, OS and HI contributed to maintenance, differentiation and immunostaining experiments using iPSC‐derived motor neurons. All authors read and approved the final manuscript.

## Supporting information


**Fig. S1.** Scatterplots of fluorescent intensity obtained from immunostainings with the thresholds. (A) Channel 1 corresponds to fluorescent intensity of Tuj1 and channel 2 corresponds to that of Islet1. The intensity threshold for channel 1 and channel 2 was set to 270 (a.u.) and 150 (a.u.), respectively. (B) Channel 1 corresponds to fluorescent intensity of Tuj1, and channel 2 corresponds to that of ChAT. The intensity threshold for channel 1 and channel 2 was set to 270 (a.u.) and 150 (a.u.), respectively. (C) Channel 1 corresponds to fluorescent intensity of eGFP (HB9), and channel 2 corresponds to that of Tuj1. The intensity threshold for channel 1 and channel 2 was set to 40 (a.u.) and 150 (a.u.), respectively.

**Table S1.** The entire list of differentially abundant mRNAs between the cells and EVs.

**Table S2.** The entire list of GO processes enriched in differentially abundant mRNAs in the cells and EVs.

## Data Availability

The generated RNA‐seq datasets in this study are available in the Gene Expression Omnibus with accession number GSE135047.
