# Supplementary figures and images for: Quantitative comparison of the mRNA content of human iPSC‐derived motor neurons and their extracellular vesicles
Source: FEBS Open Bio. 2021 Feb 2;11(2):494–506. doi: 10.1002/2211-5463.13059 (PMC7876496; doi:10.1002/2211-5463.13059)

# Supplementary Figure S1

(A)

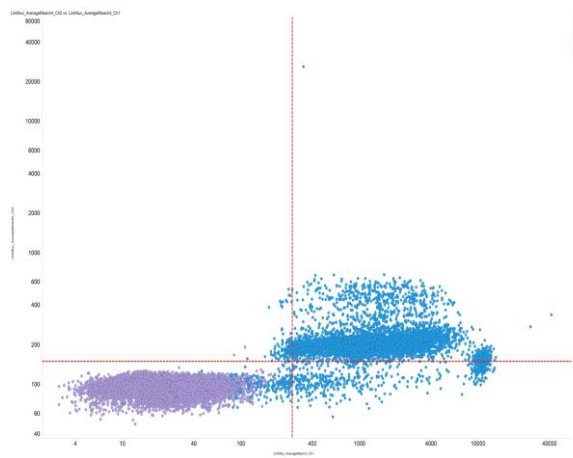

(B)

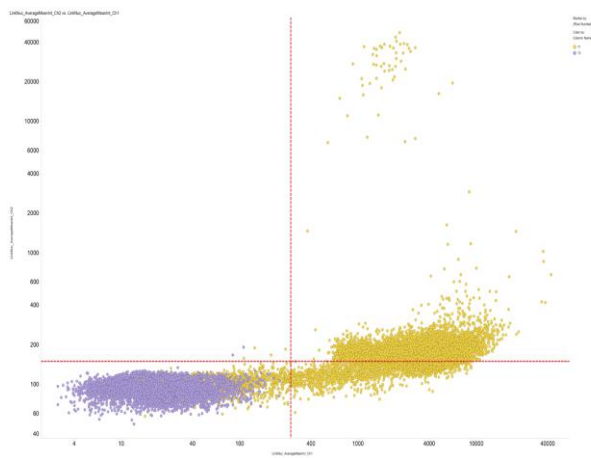

(C)

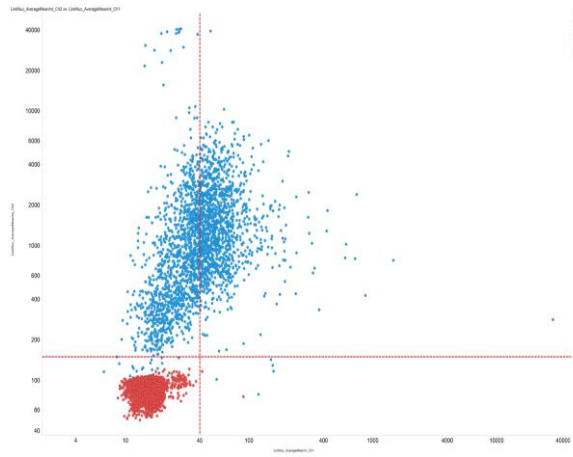

Supplement: Supplementary file 1 — Fig. S1. Scatterplots of fluorescent intensity obtained from immunostainings with the thresholds. (A) Channel 1 corresponds to fluorescent intensity of Tuj1 and channel 2 corresponds to that of Islet1. The intensity threshold for channel 1 and channel 2 was set to 270 (a.u.) and 150 (a.u.), respectively. (B) Channel 1 corresponds to fluorescent intensity of Tuj1, and channel 2 corresponds to that of ChAT. The intensity threshold for channel 1 and channel 2 was set to 270 (a.u.) and 150 (a.u.), respectively. (C) Channel 1 corresponds to fluorescent intensity of eGFP (HB9), and channel 2 corresponds to that of Tuj1. The intensity threshold for channel 1 and channel 2 was set to 40 (a.u.) and 150 (a.u.), respectively. [file FEB4-11-494-s001.pdf]
